# Improved estimation of inbreeding and kinship in pigs using optimized SNP panels

**DOI:** 10.1186/1471-2156-14-92

**Published:** 2013-09-25

**Authors:** Marcos S Lopes, Fabyano F Silva, Barbara Harlizius, Naomi Duijvesteijn, Paulo S Lopes, Simone EF Guimarães, Egbert F Knol

**Affiliations:** 1 TOPIGS Research Center IPG B.V., P.O. Box 43, 6640 AA, Beuningen, the Netherlands; 2 Departamento de Zootecnia, Universidade Federal de Viçosa, 36571-000, Viçosa, MG, Brazil

**Keywords:** Linkage equilibrium, Bootstrap, Pedigree, Genomic selection, Relationship

## Abstract

**Background:**

Traditional breeding programs consider an average pairwise kinship between sibs. Based on pedigree information, the relationship matrix is used for genetic evaluations disregarding variation due to Mendelian sampling. Therefore, inbreeding and kinship coefficients are either over or underestimated resulting in reduction of accuracy of genetic evaluations and genetic progress. Single nucleotide polymorphism (SNPs) can be used to estimate pairwise kinship and individual inbreeding more accurately. The aim of this study was to optimize the selection of markers and determine the required number of SNPs for estimation of kinship and inbreeding.

**Results:**

A total of 1,565 animals from three commercial pig populations were analyzed for 28,740 SNPs from the PorcineSNP60 Beadchip. Mean genomic inbreeding was higher than pedigree-based estimates in lines 2 and 3, but lower in line 1. As expected, a larger variation of genomic kinship estimates was observed for half and full sibs than for pedigree-based kinship reflecting Mendelian sampling. Genomic kinship between father-offspring pairs was lower (0.23) than the estimate based on pedigree (0.26). Bootstrap analyses using six reduced SNP panels (n = 500, 1000, 1500, 2000, 2500 and 3000) showed that 2,000 SNPs were able to reproduce the results very close to those obtained using the full set of unlinked markers (n = 7,984-10,235) with high correlations (inbreeding r > 0.82 and kinship r > 0.96) and low variation between different sets with the same number of SNPs.

**Conclusions:**

Variation of kinship between sibs due to Mendelian sampling is better captured using genomic information than the pedigree-based method. Therefore, the reduced sets of SNPs could generate more accurate kinship coefficients between sibs than the pedigree-based method. Variation of genomic kinship of father-offspring pairs is recommended as a parameter to determine accuracy of the method rather than correlation with pedigree-based estimates. Inbreeding and kinship coefficients can be estimated with high accuracy using ≥2,000 unlinked SNPs within all three commercial pig lines evaluated. However, a larger number of SNPs might be necessary in other populations or across lines.

## Background

In the last decades, the use of best linear unbiased prediction (BLUP) via mixed model equations [1] has allowed significant genetic progress in animal breeding programs. One of the key elements of BLUP is the use of the additive genetic relationship matrix (**A** matrix) for breeding value estimation. However, the **A** matrix may have lower accuracy due to: 1) pedigree errors and 2) inbreeding and relationship coefficients that are, almost by definition, over or underestimated. In order to derive the **A** matrix based on the pedigree, it is assumed that two full sibs from unrelated parents, have a kinship coefficient equal to 0.25. This means that they have 50% of all loci identical by descendent (IBD). However, this is not always true. Full sibs can share zero (kinship = 0), one (kinship = 0.25) or two (kinship =0.50) IBD alleles due to Mendelian sampling. Thus, actual kinship coefficients vary considerably around the expected mean [2]. Moreover, in many situations, pedigree information may not be available or incomplete, precluding the use of information from relatives.

In the last decades, new methodologies as well as software for implementation of molecular markers to estimate inbreeding and kinship have been developed [3-10]. In previous years, the availability of a limited number of markers was pointed out as the main bottleneck for the use of DNA markers for estimation of individual inbreeding using pedigree-free methods [11]. With the recent advent of high-throughput sequencing and genotyping methods, thousands of single nucleotide polymorphisms (SNPs) are now available. These offer additional opportunities for use of molecular information for estimation of inbreeding and kinship [12]. Therefore, the realized relationships can be measured more accurately using molecular markers to construct a genomic relationship matrix (**G**) [2,13].

Several studies have shown that molecular markers, such as highly polymorphic microsatellites and SNPs, are a powerful tool for verification and identification of paternity [14-18]. For a more accurate estimation of inbreeding coefficients and pairwise kinship, SNP information is used to trace back all the relationships between the animals from a given population and the individual inbreeding coefficient is based on similarity of alleles without using pedigree information.

New genotyping technologies have contributed to a reduction of genotyping costs. However, the cost of genotyping with a large number of markers is still a barrier for practical application of a **G** matrix in pig breeding programs. A reduced set of markers still able to estimate an accurate **G** matrix would contribute to substantial cost reduction of genomic selection schemes. In cattle, it was suggested that at least 2,500 preferably unlinked SNPs are needed to estimate relationship matrices [19]. In the same study, it was proposed to calibrate the number of markers to the extent of linkage disequilibrium (LD) present in the genome.

The presence of LD is an issue that has to be carefully considered for the use of dense sets of SNPs. Increasing the number of SNPs can decrease informativeness due to LD. Linked markers give more variable estimates of relatedness and inbreeding than markers in linkage equilibrium (LE). Therefore, it has been suggested that the best strategy is first to exclude tightly linked markers in order to get the most informative set [12].

The aim of this study was to optimize the selection of SNPs and determine the number of informative SNPs necessary to estimate genomic inbreeding and pairwise kinship coefficients accurately in commercial pig populations.

## Results

### Inbreeding

Summary statistics of inbreeding estimated using: all remaining SNPs after quality control (all markers), only markers in LE (LE markers) and pedigree information within the three lines evaluated (L1, L2 and L3) are shown in Table 1. The mean genomic inbreeding was higher for L2 (0.09 using all markers and 0.12 using LE markers) and L3 (0.06 using both SNP panels) compared to L1 (−0.01 using both SNP panels). However, the pedigree-based inbreeding was, on average, similar across lines (L1 = 0.03, L2 = 0.04 and L3 = 0.04). The maximum genomic and pedigree-based inbreeding coefficients were almost identical for L2 and L3, while the smallest maximal values were always observed for L1.

**Table 1:** Summary statistics of genomic and pedigree-based inbreeding estimation

	**L1**	**L2**	**L3**
**Pedigree**^**1**^			
Mean ± SD	0.03 ± 0.02	0.04 ± 0.02	0.04 ± 0.04
Minimum	0.00	0.00	0.00
Maximum	0.15	0.27	0.29
**All markers**^**2**^			
Mean ± SD	−0.01 ± 0.05	0.09 ± 0.07	0.06 ± 0.06
Minimum	−0.16	−0.13	−0.12
Maximum	0.23	0.29	0.29
**LE markers**^**3**^			
Mean ± SD	−0.01 ± 0.04	0.12 ± 0.07	0.06 ± 0.05
Minimum	−0.19	−0.14	−0.13
Maximum	0.25	0.32	0.30

Mean, standard deviation (SD), minimum and maximum values of genomic and pedigree-based inbreeding estimated within the three lines evaluated (L1, L2 and L3).

^1^Analysis performed using information from 6–10 generations recorded on paper pedigree; ^2^Analysis performed using all markers (n = 28,740); ^3^Analysis performed using only markers in linkage equilibrium (LE) within each line (9579, 7984 and 10235 LE markers for L1, L2 and L3, respectively). L1, L2 and L3 were composed of 945, 313 and 218 animals, respectively.

The average inbreeding coefficients obtained with all markers and LE markers were not remarkably different. As shown in Table 2, the Pearson’s correlations between the pedigree-based and the two genomic inbreeding values were very low (all markers: L1 = 0.30, L2 = 0.27 and L3 = 0.27; LE markers: L1 = 0.42, L2 = 0.28 and L3 = 0.35). Correlation of genomic inbreeding values with pedigree-based inbreeding increased when LE markers were used for the three lines evaluated. The correlation between the estimations using LE and all markers were >0.80 in all lines.

**Table 2:** Correlation between genomic and pedigree-based inbreeding for the three lines (L1, L2 and L3)

**L1**		**All markers**
	**Pedigree**^**1**^	
All markers^2^	0.30	
LE markers^3^	0.42	0.84
**L2**		
All markers	0.27	
LE markers	0.28	0.89
**L3**		
All markers	0.27	
LE markers	0.35	0.92

^1^Analysis performed using information from 6–10 generations recorded on paper pedigree; ^2^Analysis performed using all markers (n = 28,740); ^3^Analysis performed using only markers in linkage equilibrium (LE) within each line (9579, 7984 and 10235 LE markers for L1, L2 and L3, respectively). L1, L2 and L3 were composed of 945, 313 and 218 animals, respectively.

### Comparison of SNP panels

**Figure 1:**
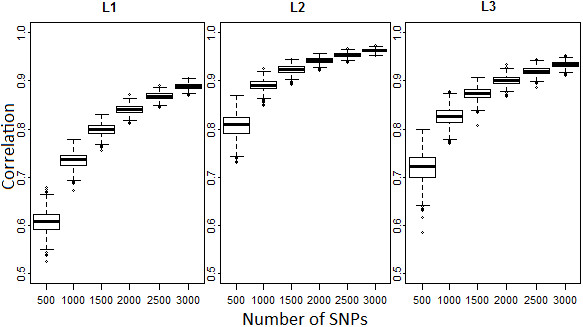
**Bootstrap analysis for inbreeding.** Box plot of correlation between the inbreeding coefficients estimated using each replicate (n = 1,000) of the subsets of LE markers and inbreeding coefficients estimated using the full set of LE markers for each line. Median is given in bold.

Figure 1 shows box plots of the correlation between inbreeding estimates using each one of the 1,000 replicates of the six panels (n = 500, 1000, 1500, 2000, 2500 and 3000 LE markers) and using the complete set of LE markers in each line. The lowest correlations were observed when 500 SNPs were used. They ranged from 0.53 to 0.68, 0.73 to 0.87 and 0.59 to 0.80 for L1, L2 and L3, respectively. Increasing the number of markers increased the correlation. When 2,000 or more SNPs were used, all replicates showed correlations >0.80 for the three lines. For all subsets, L2 showed the highest correlations, while L1 had the lowest. However, above 2,000 SNPs the additional increase in correlation was only marginal (2-5%). With 2,000 SNPs the average correlation of the 1,000 bootstrap replicates were 0.84 (L1), 0.94 (L2) and 0.90 (L3). Adding another 500 markers, the correlation for L1, L2 and L3 was on average 0.87, 0.95 and 0.92, respectively. Using 3,000 SNPs the mean correlation was 0.89 (L1), 0.96 (L2) and 0.93 (L3). Correlations between each reduced subset and all markers were comparable to those described above (data not shown) in all lines.

### Kinship

In L1, paternity testing confirmed the sire in the pedigree as the true sire for all 645 animals. Pairwise kinship between sire and offspring estimated using LE and all markers were quite similar (r = 0.83). The mean genomic kinship estimated was 0.23 ± 0.02 using both SNP panels with a range from 0.18 to 0.30 for LE markers and from 0.17 to 0.31 for all markers (Table 3). Mean pedigree-based kinship estimated between father and offspring was 0.26 ± 0.01, ranging from 0.26 to 0.33. As observed for the estimation of inbreeding, the correlation between pedigree-based and genomic kinship was higher when only unlinked markers were used (0.42 using LE markers against 0.36 using all markers).

**Table 3:** Summary statistics of genomic and pedigree-based kinship estimation

	**Father-offspring**	**Full sibs**	**Half sibs**
**Pedigree**^**1**^			
Mean ± SD	0.26 ± 0.01	0.27 ± 0.01	0.15 ± 0.01
Minimum	0.26	0.26	0.14
Maximum	0.33	0.34	0.24
**All markers**^**2**^			
Mean ± SD	0.23 ± 0.02	0.24 ± 0.04	0.12 ± 0.03
Minimum	0.17	0.08	0.02
Maximum	0.31	0.37	0.30
**LE markers**^**3**^			
Mean ± SD	0.23 ± 0.02	0.24 ± 0.04	0.11 ± 0.03
Minimum	0.18	0.08	0.02
Maximum	0.30	0.34	0.28

Mean, standard deviation (SD), minimum and maximum values of genomic and pedigree-based kinship estimated within the pairs father-offspring (n = 645), full sibs (n = 502) and half sibs (n = 5,756).

^1^Analysis performed using information from 6–10 generations recorded on paper pedigree; ^2^Analysis performed using all markers (n = 28,740); ^3^Analysis performed using only markers in linkage equilibrium (LE) within each line (9579, 7984 and 10235 LE markers for L1, L2 and L3, respectively).

Genomic kinship estimates between half and full sibs using LE and all markers also showed the same mean and standard deviation. Table 3 shows that the mean genomic kinship was equal to 0.12 ± 0.03 showing a range of 0.02 to 0.28 (LE markers) and of 0.02 to 0.30 (all markers) for half sib pair. For full sibs, the mean genomic kinship was 0.24 ± 0.04 ranging from 0.08 to 0.34 (LE markers) and from 0.08 to 0.37 (all markers). For pedigree-based kinship, half sibs showed a mean value of 0.15 ± 0.01 (0.14 to 0.24). For full sibs, kinship based on pedigree information presented a mean of 0.27 ± 0.01 ranging from 0.26 to 0.34. Correlations between pedigree-based and genomic kinship were also higher using LE markers, being 0.34 for half sibs and 0.15 for full sibs (Table 4). When all markers were used, correlations were 0.29 and 0.14 for half and full sibs, respectively. The correlations between the estimates from LE and all markers were 0.83 for kinship between father and offspring, 0.90 for full sibs and 0.92 for half sibs.

**Table 4:** Correlation between genomic and pedigree-based kinship

	**Pedigree**^**1**^	**All markers**
**Father-offspring**		
All markers^2^	0.36	
LE markers^3^	0.42	0.83
**Full sibs**		
All markers	0.29	
LE markers	0.34	0.90
**Half sibs**		
All markers	0.14	
LE markers	0.15	0.92
**Population**		
All markers	0.78	
LE markers	0.85	0.97

Correlations estimated within the father-offspring (n = 645), full-sib (n = 502) and half-sib (n = 5,756) pairs and the whole population (n = 446,040).

^1^Analysis performed using information from 6–10 generations recorded on paper pedigree; ^2^Analysis performed using all markers (n = 28,740); ^3^Analysis performed using only markers in linkage equilibrium (LE) in L1 (9,579 LE markers).

Comparing all the genomic and pedigree-based kinship pairs (n = 446,040) for the whole population of L1 animals, a much higher correlation was observed than within families. The correlation was 0.78 and 0.85 using all markers and LE markers, respectively. In this scenario, the correlation between estimates from LE and all markers was 0.97.

**Figure 2:**
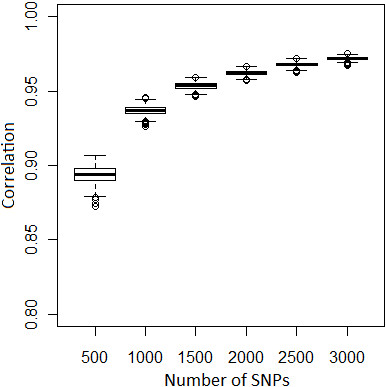
**Bootstrap analyses for kinship: Box plot of correlation between kinship coefficient estimated using each replicate (n = 1,000) of the subsets of LE markers and kinship coefficient estimated using the full set of LE markers (n = 9,579).** Pairwise kinship was evaluated only for L1 animals. Median is given in bold.

Correlations between pairwise kinship estimated using each one of the 1,000 replicates of the six subsets (n = 500, 1000, 1500, 2000, 2500 and 3000 LE markers) and the results using the complete set of LE markers are shown in Figure 2. All replicates showed correlation >0.85 with the LE markers and variation decreased with increasing number of markers in the panel.

## Discussion

### Selection of SNPs

In this study, six reduced SNPs panels were evaluated for estimation of inbreeding and kinship coefficients. The selection of SNPs was weighted by the proportion of SNPs on each chromosome in relation to the total number of LE markers. This selection procedure should ensure a good distribution of SNPs across the whole genome.

Markers located on the X chromosome were not included in the analyses because such chromosome contributes more for inbreeding in females than in males. Mendelian sampling within offspring of the same sex is lower from the male parent [12,20,21]. The population evaluated in the current work, with exception of 11 L1 and three L2 sows, was composed of boars only.

The three lines evaluated showed a different number of SNPs in the set of LE markers. Although the number of remaining markers per chromosome varied across genetic lines, the proportion of markers in relation to the total number of LE markers did not change remarkably (see Additional file 1). Therefore, for practical application, it is possible to use a mean probability (weight) to sample markers for a reduced subset for all lines simultaneously.

### Estimation of inbreeding

Inbreeding at a given locus can be defined as the probability that the two alleles from the same diploid individual in two different gametes are IBD considering a specific base population [22]. The definition of such a base population will change depending on which method has been used to estimate the inbreeding coefficients. If inbreeding is estimated based on pedigree information, the founders of the pedigree will be defined as the base population. However, if genomic instead of pedigree information is used, often the current population is defined as the base population [22]. This represents a convenient strategy because most of the modern livestock genetic lines were generated decades ago and genetic material from the founders is not available for genotyping.

In the current study, the current population was defined as the base population. Therefore, using allele frequencies estimated from the actual population, the correlation between genomic and pedigree-based inbreeding using LE markers ranged from 0.28 to 0.42 and 0.27 to 0.30 using all 28,740 markers whereas correlations between LE markers and the full set of markers ranged from 0.84 to 0.92 (Table 2). Low correlation (r < 0.25) between genomic and pedigree-based inbreeding coefficients were also reported in simulation study in humans [23]. VanRaden [9] obtained even negative correlation between genomic and pedigree-based inbreeding (−0.26 to 0.40) using actual allele frequencies estimated by counting alleles in the genotyped population. Higher correlation between genomic and pedigree-based inbreeding have been observed when the allele frequency of the founders of the pedigree was used (i.e. genomic and pedigree-based inbreeding were estimated using the same base population). Using simulated genotypes, VanRaden [9] has shown that the correlation between genomic and pedigree-based inbreeding ranged from 0.78 to 0.81 and from 0.66 to 0.75 when the allele frequencies in the base population (founders of the pedigree) were known or estimated, respectively. VanRaden et al. [21] observed that including all available 43,385 SNPs, the correlation between genomic and pedigree-based inbreeding ranged from 0.50 to 0.56 when the allele frequencies of the base population (founders of the pedigree) were estimated, and increased up to 0.59 – 0.68 when an allele frequency of 0.50 was used.

Therefore, the low correlation between genomic and pedigree-based inbreeding in this study can be partly explained by the fact that different base populations were adopted for each method. In addition, genomic inbreeding in this study might be underestimated because markers with a minor allele frequency (MAF) <0.05 were excluded and the populations investigated are related to the main breeds used for SNP detection and selection for the 60K SNP array [24]. In any case, the true inbreeding is not known. Other tests, rather than a higher correlation between genomic and pedigree-based inbreeding, need to be established because pedigree information neglects Mendelian sampling variance and only covers inbreeding of the most recent generations. However, although it is clear that the pedigree-based inbreeding cannot be taken as a golden standard, the comparison between genomic and pedigree-based estimates was performed in this study to make it comparable to those results from the literature [9,21,23].

A larger mean of genomic compared to traditional inbreeding may also be expected because selection increases the probability for favorable alleles to be transmitted to later generations [21,25]. This is reflected by the higher values of genomic inbreeding compared to pedigree-based inbreeding. Only for L1, the mean genomic inbreeding was lower than the pedigree-based inbreeding and even slightly negative (−0.01). Negative values for inbreeding indicate that the animal is less homozygous than expected [22]. In cattle, VanRaden et al. [21] observed negative values for all three breeds that they evaluated. As expected, in the current study, the standard deviation (SD) of the genomic inbreeding was higher than the SD of the pedigree-based estimate for all lines. The difference in SD is due to the fact that genomic information measures homozygosity of each individual instead of the mean homozygosity expected from common ancestors. It has been discussed that in a future involving genomic selection the pedigree-based inbreeding may not be a valid inbreeding measurement due to underestimation of the true inbreeding [25].

### Estimation of kinship

Paternity of animals could be checked only for animals whose fathers were also genotyped. Due to the absence of genotypes of the dams, only the relationship between father and offspring was investigated. Nevertheless, it is necessary to keep in mind that pedigree-based kinship was estimated based on information that ranged from 6 to 10 generations and, even in accurate systems such as the one used in this study, mistakes can occur at information recording or introduction into the database generating pedigree mistakes. If the on-farm pedigree is not correct, the correlation between pedigree-based and genomic kinship will be affected too.

**Figure 3:**
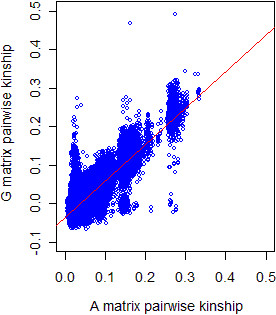
**Plot of G vs. A pairwise kinship.** Plot of genomic kinship **(G)** using the full set of LE markers (n = 9,579) against the pedigree-based kinship **(A)** for all pairwise combinations among all 945 L1 animals (n = 446,040). Trendline is given in red.

Higher correlations between the genomic and pedigree-based methods were observed when the full population was evaluated compared to within-family analyses. However, the average and the SD of pairwise kinship between father-offspring and full and half sib pairs were practically the same (Table 3) using both sets of markers. In the current study, the correlation between genomic and pedigree-based kinship for the whole population was 0.85 using LE markers and 0.78 using all markers. This is comparable to the studies of Rolf et al. [19] and Pimentel et al. [26], where genomic pairwise relatedness among individuals from their cattle populations showed a correlation with pedigree-based relatedness equal to 0.87 and 0.73, respectively. Such a high correlation is expected because when the whole population is analyzed, kinship between many unrelated animals is estimated. Doing so, both genomic and pedigree-based kinship are close to zero (Figure 3), which results in a high correlation between both estimates.

Figure 3 shows all pairwise combinations between **G** and **A**. It can be observed that two individuals have a genomic pairwise kinship greater than 0.45. This means that these animals share more than 90% of their genotypes, so they must be monozygotic twins or mistakes from either the sample collection or the DNA extraction (same animal sampled twice and identified with different ids). Moreover, a group of animals registered as full sibs in the on-farm pedigree (pedigree-based kinship above 0.25), showed a genomic pairwise kinship around zero. Although it is possible that two full sibs have a kinship coefficient around zero, such extremes are not expected. Thus, those observations are a clear indication of on-farm pedigree mistakes. This shows how the **G** matrix can be used as an important tool for recovering on-farm pedigree mistakes.

The expected kinship coefficient for father-offspring pairs is at least 0.25 (mating an unrelated sire and dam) because the offspring inherits 50% of the genetic information from the sire. In the current work, the mean genomic pairwise kinship for father-offspring was 0.23 ± 0.02 (Table 3) which was somewhat lower than expected. It is important to highlight that expected pedigree-based kinship is based on IBD and the genomic kinship here estimated is based on identical by state (IBS). IBD allele refers to alleles that are the same from a common ancestor in a base population, while IBS simply refers to alleles that are the same, irrespective of whether they are inherited from a recent ancestor [22]. Moreover, IBD alleles are an unobservable quantity, while what can be observed is the allele state, alleles that seem to be the same (i.e. IBS alleles) [27].

Another possible reason for the lower genomic kinship estimate for father-offspring pairs is segregation distortion. This refers to a phenomenon responsible for a significant deviation of the observed allele frequencies compared to the expected frequencies under Mendelian segregation [28]. In a recent study, Zhan & Xu [29] described that segregation distortion seems to be more common than expected.

In addition, it is important to keep in mind that the genomic relationships estimated in this study might contain sampling error due to the finite number of SNPs used. As discussed by Powell et al. [22], it is difficult to define the base when single loci across the genome are used to estimate the relationship between individuals of a given population. Consequently, the method can fail to take distant relationships into account. This sampling error may be another explanation why the kinship between father and offspring was lower than expected (<0.25) (Table 3). Moreover, the sampling error can also partly explain that many animals with a low pedigree-based relationship do not show any relationship based on the genomic measurement (Figure 3). A methodology able to estimate relationship using all SNPs simultaneously has been proposed as a solution to the biased relationship estimates [30,31].

**Figure 4:**
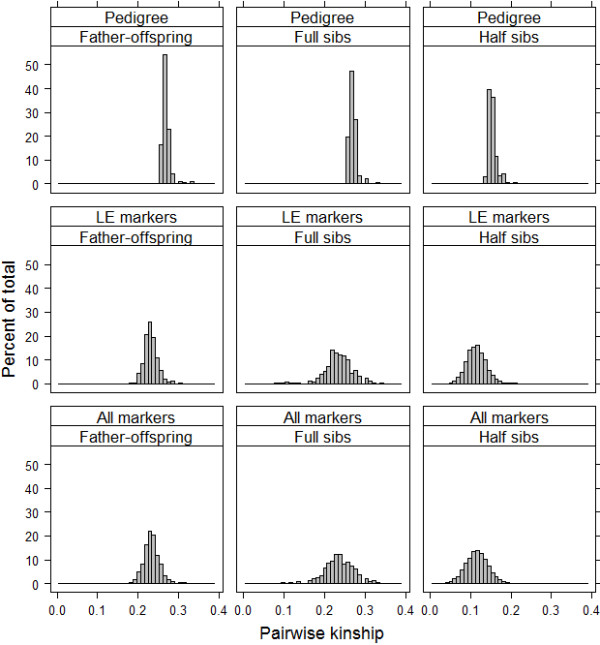
**Distribution of genomic and pedigree-based kinship.** Genomic kinship was estimated using all markers (n = 28,740) and LE markers (9,579). Pedigree-based kinship was estimated based on 6 up to 10 generations. Pairwise kinship between 645 father-offspring pairs, 502 full sibs pairs and 5,756 half sibs pairs was evaluated.

For full sibs, the same pedigree-based kinship coefficients are expected if parents are unrelated (0.25). However, a large range in the genomic estimate of kinship was observed (Figure 4). These results fit our expectations due to the fact that full sibs can share zero, one or two IBD alleles at each locus. If two full sibs share two alleles IBD for all loci, the pairwise kinship will be 0.50. But, if they share zero alleles IBD, the kinship will be zero. Therefore, a variation around the average is expected. In a study evaluating the variation in the real relationship of 4,401 pairs of human full sibs [2], an average pairwise kinship of 0.25 was estimated, ranging from 0.19 to 0.31. In the current study, a larger variation was observed. The mean genomic pairwise kinship was 0.24 (ranging from 0.08 to 0.34 when LE markers were used). For half sibs, the pedigree-based kinship expected is at least 0.125 and in the current study the coefficients ranged from 0.02 to 0.28 with average of 0.12 when LE markers were used (Table 3 and Figure 4).

### Reduced SNP panels

Genomic inbreeding and kinship achieved higher correlations with pedigree-based measurements when LE markers instead of all markers were used. However, the mean and standard deviations values of genomic kinship and inbreeding were practically the same using both sets of SNPs (Tables 1 and 3). The correlations between inbreeding and kinship coefficients estimated in all different scenarios using LE and all markers ranged from 0.83 to 0.97 (Tables 2 and 4).

As outlined above, for father–offspring pairs, the expected value of the genomic kinship of father-offspring pairs is 0.25 for every individual pair if sire and dam are unrelated. However, in livestock species, there is always some relatedness between parents originated from the same population. Thus, the kinship between father and offspring is expected to be higher than 0.25. But a large variation above this value is not expected. Therefore, the standard deviation of the genomic kinship of father-offspring pairs obtained using the set of LE markers can be used in comparison with the full set of markers as a parameter to measure the accuracy of the genomic kinship rather than the correlation with pedigree-based kinship. This standard deviation does not differ between LE markers and using all markers (Table 3) showing that both sets of markers achieve comparable accuracy.

Moreover, both sets of markers were able to better capture the variation of kinship between sibs due to Mendelian sampling compared to the pedigree-based method (Figure 4). Previous studies have reasoned that increasing the number of markers may not result in any appreciable increase of information [12] and that the genomic relationship matrix has to be estimated ideally with unlinked markers [19]. Another study [27] argues that the large number of SNPs is partly illusory due the fact that an increased marker density implies increased dependencies due to LD. The results of the present study confirmed that decreasing the number of markers didn’t result in reduction of information. Thus, using a reduced set of markers in LE it is possible to estimate accurate genomic inbreeding and kinship coefficients and makes it possible to reduce genotyping costs as well.

For kinship estimation a lower number of markers was needed than for inbreeding estimation. The subset with only 500 markers achieved a high correlation (>0.85) with the results obtained using all LE markers. Moreover, the variation across the results of each replicate was smaller (Figure 2). However, subsets <2,000 SNPs could not be used for breeding value estimation because inbreeding coefficients would not be estimated accurately.

**Figure 5:**
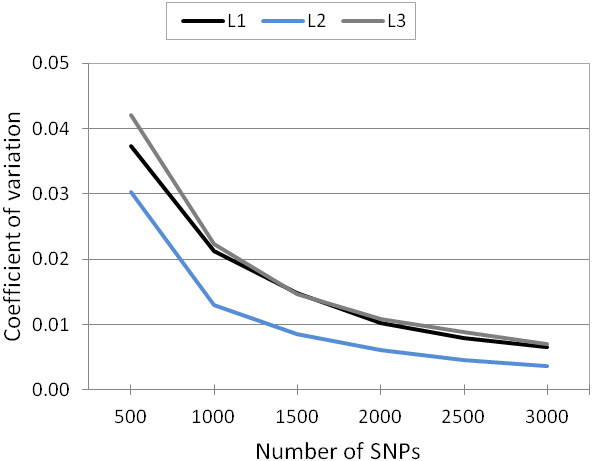
Coefficient of variation between replicates (n = 1,000) of the subsets of LE markers for inbreeding estimation.

For inbreeding, increasing the number of markers above 2,000 per line only resulted in a small increase (<5%) of the mean correlation across the subsets and the full set of LE markers (Figure 1). Comparing the coefficients of variation (CV) across the correlation of each replicate of the six subsets and the full set of LE markers, the CV fell below 0.01 for all lines using 2,000 or more SNPs (Figure 5). The absence of large variation across replicates suggests that it does not matter which unlinked markers were sampled. The highest correlations between the marker subsets and the LE markers as well as the lowest CV were observed in L2. This is expected as L2 also had the highest inbreeding coefficient. To analyze inbreeding only for L2 animals, 1,500 SNPs should be sufficient. However, for practical applications, it is not feasible to create different sets of markers for each breed or line. It is important for the breeding companies to establish a common set of markers that can be used for all their different lines.

The first step to define a consensus set is to identify a set where all SNPs are polymorphic in all lines. This means that MAF has to be calculated within line, and only the SNPs that achieve the desired threshold in all lines go to the next check. The second step is to investigate the LD also within line, and afterwards to exclude tightly linked markers. Different lines will present different extensions of LD across the genome. However, they will also share LD blocks. Then, the last step before defining a consensus set is to keep the same representative SNP of each shared block for all lines. The consensus set could be used across lines if this set contains ≥2,000 unlinked SNPs. If several lines are used, it might be difficult to define a set with only 2,000 – 3,000 SNPs due to the difference in LD and allele frequency across lines. This difference in LD is well described by Ai et al. [32] comparing LD extent in Western and Chinese pig populations. These authors have shown that LD (r^2^ ≥ 0.3) in an admixed Western pig population (White Duroc) was much larger than in the Chinese Wild boar (750 and 38 kb, respectively). The inter-population LD extent (r^2^ ≥ 0.3) was of 125 kb across Western pigs and 10.5 kb across Chinese pigs [32].

In this study, after evaluating the group of LE markers for each of the three lines, only 1,046 common SNPs were found. However, the lower number of common SNPs is related to the random exclusion of linked markers within lines performed by PLINK software. Different SNPs from the same LD block might have been selected for different lines. A larger number of common SNPs is expected if the same representative SNP of each shared block for all lines is selected.

In the current study it was shown that ≥2,000 unlinked SNPs within a pig line enabled the estimation of inbreeding and kinship coefficients with high accuracy. In cattle, Rolf et al. [19] have shown that 2,500-10,000 SNPs were needed for robust estimation of genomic relationship matrices. With 10,000 SNPs the genomic relationship coefficients seemed to be extremely robust while building the **G** matrix with 2,500 SNPs seemed to be very sensitive to SNP sample size. The reduced sets of SNPs analyzed by Rolf et al. [19] were, however, randomly sampled across the genome, ignoring the LD between the selected SNPs, thus potentially overestimating the number needed. Moreover, the requirement of a lower number of SNPs to build a **G** matrix for pigs compared to cattle can be explained by the difference in genetic length of their genomes and the length of their haplotype blocks. Tortereau et al. [33] observed that the total genetic map length in pigs varied from 1,797 to 2,149 cM. In cattle, it was shown that the genetic length of the genome was 3,249 cM [34]. In addition, Veroneze et al. [35] showed that greater block size was observed in commercial pig populations compared to cattle. Working with 6 pig populations genotyped using the Illumina Porcine SNP60K BeadChip, Veroneze et al. [35] estimated that the average block size was 395 kb. For a Holstein–Friesian cattle population genotyped using the Illumina Bovine SNP50K BeadChip, Qanbari et al. [36] estimated that the average block size was 164 kb.

In order to perform a final examination on the number of markers needed for the genomic relationship matrix, it is necessary to evaluate the power of the approach in practical application for estimation of breeding values. Subsets of ≥2,000 unlinked SNPs within line are expected to yield results in agreement to those presented in a recent study [37] using real and simulated data from a sheep population. Clark et al. [37] have shown that breeding values are estimated with higher accuracy using the full set of SNPs (50K SNP ovine SNP chip) via genomic BLUP instead of using the pedigree-based relationships via BLUP. Increased accuracy of breeding values estimation due to use of genomic relationships has also been reported by Hayes et al. [30].

## Conclusions

The present study shows that variation of kinship between sibs due to Mendelian sampling is better captured using genomic information than the pedigree-based method. Therefore, the reduced sets of SNPs could generate more accurate kinship coefficients between sibs than the pedigree-based method. Variation of genomic kinship of father-offspring pairs is recommended as a parameter to determine accuracy of the method rather than correlation with pedigree-based estimates. Inbreeding and kinship coefficients can be estimated with high accuracy using ≥2,000 unlinked SNPs within all three commercial pig lines evaluated. However, a larger number of SNPs might be necessary in other populations or across lines. A genomic relationship matrix estimated using unlinked markers will be further tested for estimation of breeding values to validate the methodology.

## Methods

### Animals

This experiment was conducted strictly in line with the Dutch law on the protection of animals. A total of 1,565 animals from three commercial pig lines (Duroc-based L1 n = 1,008, Large White composite L2 n = 316, and Pietrain-based L3 n = 241) were genotyped. With the exception of 11 L1 and three L2 sows, all evaluated animals were boars. The structure of the population was as follows: L1 consisted of 628 offspring with only the father genotyped, 26 offspring with only the mother genotyped and 69 had both parents genotyped. In total, 41 sires and 11 dams were genotyped for L1. For L2, 49 offspring had only the father genotyped, while two had only the mother and one with both parents genotyped, resulting in a total of seven sires and three dams also genotyped. In L3 only one family was genotyped (8 offspring and one sire). No direct parent-offspring connection existed for the remaining animals from this population.

### Selection of SNPs

Genotyping was performed using the PorcineSNP60 Beadchip of Illumina (San Diego, CA, USA) [24]. All animals were genotyped for 64,232 SNPs at Service XS (Leiden, The Netherlands). After quality check, 10,210 SNPs were removed because of low quality score (GenCall score <0.7). A threshold of 30 pedigree errors or more was applied and 190 SNPs were removed. In addition, 20,736 SNPs were excluded from analyses due to MAF < 0.05 in at least one of the three lines. An additional 374 markers with a call rate < 95% were also excluded. A total of 3,982 located in the one of the sex chromosomes were also excluded. More details about DNA preparation and genotyping process can be accessed at Duijvesteijn et al. [38].

With respect to animals, 89 animals (L1 n = 63, L2 n = 3 and L3 n = 23) were excluded due to frequency of missing genotypes > 5%. For further analyses, 945 (L1), 313 (L2) and 218 (L3) animals remained with genotypes for a total of 28,740 SNPs (all markers) spread across the 18 autosomes (build9). MAF, call rate and missing genotype frequency were estimated using PLINK software [8].

Subsets of the most informative markers for each line were selected based on estimates of LD between SNPs. Tightly linked SNPs were then excluded using LD-based SNP pruning in PLINK, creating a group of LE markers. LD was estimated between each pair of SNPs in a window of 50 SNPs. If the LD was greater than 0.5 (r^2^ > 0.5) one of the pair of SNPs was removed and the window shifted five SNPs forward. The procedure was repeated until the end of each chromosome was reached.

Finally, to evaluate the number of markers needed for a reduced panel of SNPs, six panels with different number of markers were created from the LE markers (n = 500, 1000, 1500, 2000, 2500 and 3000). Each panel was replicated with replacement 1,000 times using the bootstrap procedure in R [39]. Selection of the number of SNPs for each panel was weighted according to the number of markers available on each chromosome, in relation to the total number of LE markers. This ensures that chromosomes with larger number of available markers have more SNPs represented in the reduced panel. Thereby, each chromosome had the same proportion of markers sampled in the different subsets.

The number of markers present in the set of LE markers after LD based SNP pruning (9,579, 7,984 and 10,235 for L1, L2 and L3, respectively) and the proportion of markers remaining for each chromosome in relation to the total number of markers [see Additional file 1]. The average spacing between markers in the set of LE markers was 0.22, 0.27 and 0.21 Mb for L1, L2 and L3, respectively.

### Inbreeding and kinship estimation

Kinship or co-ancestry coefficient represents the probability that two genes, sampled at random from each individual are identical (e.g. the kinship coefficient between a parent and an offspring is 0.25). It equals half of the numerator relationship or coefficient of relatedness [40,41]. The inbreeding coefficient is the kinship coefficient between the individual’s parents, and measures the probability that an individual has a pair of alleles that are identical by descent from a common ancestor.

Genomic inbreeding was estimated for all the three lines separately. Kinship coefficients were estimated only for L1 animals since it had the largest number of genotyped sibs and parents-offspring pairs. Pairwise kinship between the father-offspring, full sib, and half sib pairs were estimated only for animals for which the father was also genotyped and the paternity was confirmed by DNA analysis (n = 645). Paternity verification was conducted using the panel of 120 SNPs and criteria proposed in a recent study [18] with the program CERVUS [42].

Genomic inbreeding and kinship coefficients were estimated using (1) all markers, (2) LE markers and (3) each replicate of the six panels, using the IBS function of the package GenABEL [10] using R.

Calculation of genomic inbreeding and kinship was weighted by the allele frequency within each line. GenABEL calculates a **G** matrix based on average IBS. The coefficient of IBS (F) for a pair of individuals *i* and *j* was computed as follows:

$$F_{\mathrm{ij}}=\underset{k}{\sum }\frac{\left(x_{i,k}-p_{k}\right)*\left(x_{j,k}-p_{k}\right)}{\left(p_{k}*\left(1-p_{k}\right)\right)}$$

where *k* ranges from 1 to N = number of SNPs, *x*_*i,k*_ is a genotype of *i*^th^ individual at the *k*^th^ SNP, coded as 0, 1/2, 1, corresponding to the homozygous, heterozygous, and other type of homozygous genotype and *p*_*k*_ is the frequency of the allele that has been coded as 1 [10]. Individuals which are more heterozygous than expected based on the allele frequency of the population show a negative inbreeding coefficient indicating that they are less homozygous than expected for a population under Hardy-Weinberg equilibrium.

In order to compare molecular and pedigree-based relationship estimation, the program ENDOG v4.8 [43] was used to estimate the **A** matrix based on pedigree information from 6 up to 10 generations. The inbreeding estimated by ENDOG was defined as the probability that an individual has two identical alleles by descent [43] and was computed following Meuwissen & Luo [44].

## Competing interests

The authors declare that they do not have any competing interests.

## Authors' contributions

MSL conducted statistical analyses, prepared figures and tables and wrote the manuscript. FFS was involved in the statistical analyses and reviewed the manuscript. BH was involved in discussion on statistical issues and writing of the manuscript. ND was involved in sample collection, organization of the genotyping experiment and writing of the manuscript. PSL was involved in the statistical analysis and general discussion of the results. SEFG and EFK were involved in planning the project, statistical supervision and experimental set up. All authors have read and approved the final manuscript.

## Supplementary Material

Additional file 1 **SNPs per chromosome.** Number and proportion of SNPs from each chromosome (Chr) in the set of LE markers for all lines evaluated (L1, L2 and L3). Click here for file
